# Flexible modular dwelling for rural environments; specific case: Cebadas – Ecuador

**DOI:** 10.12688/f1000research.154417.2

**Published:** 2025-01-17

**Authors:** Karina Elizabeth Cajamarca Dacto, Jean Carlos Montero Riofrio, Erick Fabricio Nieto Páez, Dany Marcelo Tasan Cruz, Maikol Josueé González Espinosa

**Affiliations:** 1 Faculty of Engineering, Universidad Nacional de Chimborazo, Riobamba, Chimborazo Province, Ecuador; 2 Higher Technical School of Construction, Universidad Politecnica de Madrid, Madrid, Spain

**Keywords:** Housing, sustainability, rural, design, modular, flexible, bioclimatic, adaptable

## Abstract

**Background:**

Sustainable modular dwelling design for rural areas should focus on creating healthy and economically accessible spaces, sensitive to local needs and integrating environmental, functional, sociological and technological aspects. Flexibility is essential to reduce the initial investment and allow future transformations, optimizing the recovery and reuse of materials. These houses must be adaptable, safe and have basic services, satisfying the needs and stages of families’ development. In Ecuador, poverty and energy inefficiency worsen the quality of life in rural areas. This project in the Cebadas parish proposes modular dwelling that applies bioclimatic and sustainable criteria, using local materials to improve habitability and promote the social and economic development of the community.

**Method:**

The research approach is qualitative-quantitative. Qualitative, since it is necessary to identify the qualities of the sector, bibliographic exploration of guidelines and sustainable strategies. Quantitative, to evaluate the degree of affectation through a diagnosis of the study site and to provide efficient solutions that respond to the context and social reality.

**Results:**

The spaces presented in the proposal respond to the spatial need for growth and expansion of the users of the sector, through 6 basic guidelines that this type of dwelling should have: Environment, Visual, Form, Function, Bioclimatic and Materials.

**Conclusions:**

The flexible modular dwelling project for rural environments addresses complex housing needs, highlighting the flexibility and adaptability of an expandable module. It includes income generation with a duplex apartment, fosters communal and cultural life with public and commercial spaces, promotes tourism and rural development with landscape design and crops. In addition, it ensures sustainability through bioclimatic strategies and responsible use of resources.

## 1. Introduction

As part of sustainable design in the creation of healthy spaces, economically viable and sensitive to local needs, Garzón (2010), mentions that “Buildings should be designed and constructed in such a way that their purposes (environmental, functional, sociological and symbolic aspects) are interrelated and achieved through their means (technological and morphological aspects) in order to allow their habitability, operation and maintenance with the efficient use of natural and cultural resources of the area and with low levels of dependence (energy, economic, etc.) to minimize the impacts on their contexts. This is how the term flexibility is born as a viable option for rural housing, since it allows reducing the initial investment and facilitates the dwelling to be transformed, improved and completed over time, according to the needs, possibilities and preferences of the household members in a sustainable way.

Sanchez (2011) states that sustainable design refers to buildings that allow managing their end-of-life cycle efficiently. It ensures that the disassembly of the building reduces waste generation and maximizes the recovery of its high-value components, as well as materials for reuse and recycling. This process should encourage builders to design structures that are flexible in order to ensure the efficient operation of the construction, its maintenance, as well as its dismantling; allowing a variety of future scenarios for the building.

The key to achieve a truly flexible and adaptable dwelling is the ability and ease to recover the components and materials that make it up. Much of the current difficulties with deconstruction are due to the design inflexibility and unsuitability, as well as the demands of new requirements. Therefore, existing buildings at the end of their life cycle should be seen as new sources for new constructions, and for this, it must be understood that spatial systems cannot be treated independently from technical ones, since spatial transformations are directly related to the technical composition of the building. Consequently, spatial flexibility can be defined as: Spatially extensible, Divisible to reconfigure its space, Multifunction within the same space. Also, technical flexibility is related to the ease for: interchangeability, displacement, reuse and recycling of the elements and systems that define the structure such as: accessibility, replacement, reconfiguration and separation.

Therefore, buildings should be able to adapt to new trends, adopting new technological advances and facilitating the updating of its elements and systems, that is to say that they must be designed to adapt and deconstruct; creating sustainable buildings means designing to build demountable structures, maximizing their flexibility both in their spatial configuration and in having a lesser impact on the environment; this will be achieved by taking into account their complete life cycle, which goes from extraction to the end of their use, that is, their manufacture, design, construction, use and renovation. Hence, sustainable design is established as a combination of design strategies that address the same problem at different scales. These approaches are: design to recover, to reuse, to be durable, to use materials efficiently, to reduce waste, to regenerate, to repair, to adapt. In order to formulate an adaptive strategy, it is necessary to define what is sustainable housing, which can be defined as the habitable enclosure containing the minimum components to house a family nucleus. This basic unit, in an adaptive process such as the one we are trying to understand here, is an initial phase understood as a structure that is delivered unfinished, and with time, consolidates into a complete dwelling, this initial phase corresponds to the basic unit with maximum space and minimum cost, to house human activities in an adequate manner. For this, it must comply with some basic characteristics translated both in spaces and services.

Housing must be stable (safe) and have basic household services. In addition, it must differentiate spaces to properly house the different activities that take place within, “the four vital activities: rest, food preparation, sanitary activities, as well as family and cultural development gatherings” ( Moncaleano & Morales, 2006). Not only does each family nucleus have different needs, but the stages of a family’s development over time must also be taken into account. The stages of household development must be attended to simultaneously. A consolidated household with children needs a complete dwelling or one in the process of being so; on the other hand, a newly couple may only need a rented space for the first stages of family consolidation; a flexible modular dwelling must consider the evolutionary factor in the organization of the different spaces that make it up.

Addressing the issue of housing requires a more comprehensive approach, focusing on interculturality and social, economic and environmental sustainability, hence it is essential to consider access to housing within the framework of rural development proposals that include support for production, implementation of marketing circuits, inclusion of new productive alternatives such as ecotourism or community tourism, development of energy technologies, strengthening of the social fabric and intercultural coexistence, among others.

The problem of self-construction and informality in Ecuador are only one aspect of the social problem of housing. Energy inefficiency and the high carbon emissions generated by construction increase the environmental impact of housing, as well as the existing poverty of the sectors, which together cause unsustainability and inefficiency. According to Pinto and Ruiz (2009) one out of every three Ecuadorian families lives in rural areas, where despite the fact that the population growth rate is three times lower than in the urban population, they concentrate the highest poverty rates. As a result, it is estimated that 4 out of every 5 families in rural areas are poor, which is evident in the quality of housing. It is estimated that more than 80% of them do not meet the habitability conditions necessary to ensure the life quality of families, due to lack of services, poor quality construction, overcrowding and insecurity. In the case of housing in Cebadas, one of the poorest parishes in Ecuador, 80.19% of the houses do not have acceptable living conditions, affecting the quality of life and development of the people.

Consequently, this research aims to suggest an architectural proposal for flexible modular dwelling in the Cebadas parish of Guamote canton in the province of Chimborazo, in response to the spatial requirements for growth and expansion presented by the inhabitants of the sector through strategic ideas that address bioclimatic criteria, modular design, using local materials, thus establishing principles of sustainability to be considered in future buildings, so that they are projected as sustainable, flexible housing.

## 2. Methods

### 2.1 Research design

The research is qualitative-quantitative; qualitative, since it is necessary to identify the qualities of the area, bibliographic exploration of 6 basic guidelines that this type of housing should have: Environment, Visual, Form, Function, Bioclimatic and Materials that will help to generate a solid base for the sustainable proposal. Quantitative, to evaluate the degree of affectation through a diagnosis of the study site and provide efficient solutions.

### 2.2 Type of research

This research aims to raise an architectural proposal of flexible modular dwelling in the Cebadas parish of the Guamote canton in the province of Chimborazo, which responds to the spatial need for growth and expansion presented by the inhabitants of the sector, it will also allow to solve current and future problems of sustainable criteria in buildings. Therefore, the ideal is to use a mixed approach, since it will allow obtaining necessary and essential qualitative data for the analysis of the sector, allowing to generate a broad vision on the critical thinking about architectural design, construction, standard of living, and quality of materials in the sector. Quantitative, for the collection of data, to have explicit values about the spatial needs in housing, as well as their functional and bioclimatic quality; in order to generate a proposal that addresses all these issues to solve them with a sustainable and flexible architectural design.

### 2.3 Level of research

Exploratory and propositional. The exploratory type identifies the problems affecting the area under study, which will provide data and diagnostic results including the bioclimatic requirements of exteriors and interiors. Propositional is the one that forces the previously developed research to provide ideas for an architectural proposal as well as sustainable strategies to solve the previously identified problems.

### 2.4 Research modality

Applied or Practical, since its intention is to improve the quality of life in the Cebadas parish in the province of Chimborazo and contribute to the construction of new knowledge in the field of rural housing sustainability.

### 2.5 Methodology

The research, qualitative-quantitative; qualitative, since it is necessary to identify the qualities of the area at the macro level, bibliographic exploration of basic guidelines that this type of housing should have: Environment, Visual, Form, Function, Bioclimatic and Materials that will help to generate a solid base for the sustainable proposal. Quantitative, to evaluate the degree of affectation through a diagnosis of the study site and provide efficient solutions in a flexible modular housing proposal for the Cebadas parish of the Guamote canton in the province of Chimborazo.

### 2.6 Research procedures and techniques

Field studies will be applied to diagnose the current state of the area. For data collection, the bibliographic research technique will be used to gather information about the cosmovision, traditions and available materials, as well as the existing problems in the study area by the inhabitants of the place. Once the information is obtained, it will be analyzed to design a plan according to the situation and needs of the sector.

### 2.7 Study population

Due to the territorial extension of the Cebadas parish, it has been considered to establish the research in Zone 2, in the parish head of Cebadas. Initially, it is planned to obtain information from the Decentralized Autonomous Government of Pichincha and the Development and Land Use Plan of Cebadas, as well as from the critical observation of the site visit, and then conduct a diagnosis of the place to obtain more accurate data on the needs of the sector.

## 3. Results

### 3.1 Diagnosis

Following the suggested methodology, the analysis of the houses in the study sector revealed that most of them are in a state of deterioration or in ruins. The evident precariousness of the sector prevents the maintenance or improvement of the houses, generating insecurity because of the deterioration of the structure, leading to disuse and abandonment.

Regarding the function of the houses, it is important to emphasize that they present: spaces whose dimensions do not respond to the needs of their inhabitants, lack of elements of direct connection with the immediate environment; such as large windows or balconies; poorly lit and uninhabitable spaces, lack of consideration in the future projection of housing, as well as lack of adaptability and flexibility of the spaces. All this has caused the abandonment of the houses, absence of spaces for production and breeding, and lack of bioclimatic and sustainability strategies.

In the formal approach, it should be noted that those dwellings that have undergone changes to adapt to the new conditions of the users have suffered formal affectations. Meanwhile, in the bioclimatic aspect, it is evident that despite the fact that the buildings are constructed with materials of high thermal inertia, the lack of lighting and the use of passive air conditioning systems have caused the internal spaces to be cold and unwelcoming.

Therefore, in the second stage an analysis of the bioclimatic requirements for outdoor and indoor spaces in the study area is carried out, to make informed decisions during the design, in order to optimize both thermal comfort and energy efficiency.

**Figure 1.**
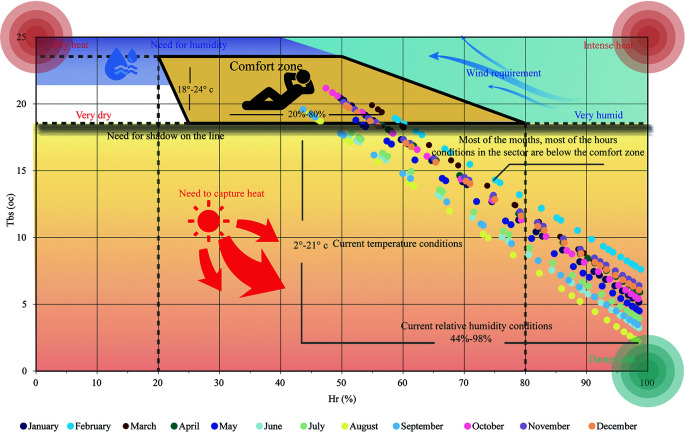
Analysis of the Bioclimatic as from of Olgyay (1963) applied to Cebadas using Microsoft Excel and Adobe Illustrator.

Figure 1 shows the bioclimatic chart of Olgyay (1963), applied to Cebadas. It shows a comfort zone that includes the ideal temperature and humidity values for outdoor environments. The ideal temperature values range between 18 and 24 degrees Celsius, and the relative humidity between 20% and 80%. In the case of Cebadas, most of these points are outside the comfort zone, presenting temperature ranges from 2 to 21 degrees Celsius and relative humidity from 81% to 100%. This indicates that heat is required during all months of the year, highlighting the need to use materials, colors and textures with high thermal inertia to absorb heat and prevent heat loss.

**Figure 2.**
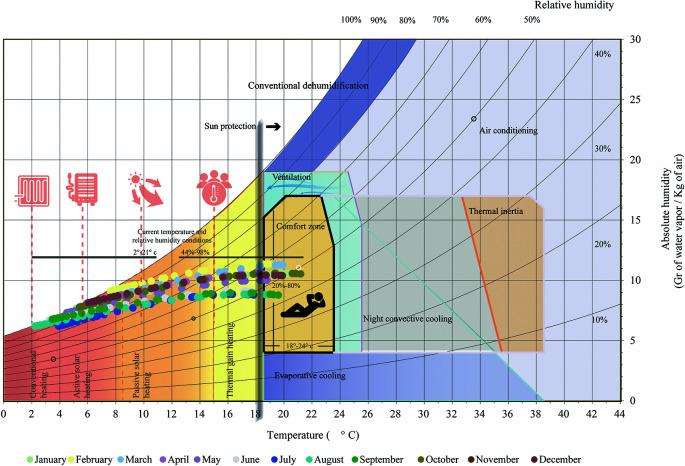
Analysis of the Bioclimatic Chart as from of Givoni (1969) applied to Cebadas using Microsoft Excel and Adobe Illustrator.

In the case of bioclimatic requirements for exteriors, it is noted that, above 18.5 degrees Celsius, solar protection is necessary to avoid the direct incidence of solar radiation, combined with other strategies to protect the interior of the building. As shown in Figure 2:

• Conventional heating: for temperatures below 2.5 degrees Celsius, coal, gas or electric power is required.
• Active solar heating: For temperatures between 2.5 and 8.5 degrees Celsius, support systems such as pumps and fans are required.
• Passive solar heating: For temperatures between 8.5 and 15 degrees Celsius, systems that passively capture and distribute solar energy are used.
• Internal gain heating: For temperatures between 15 and 21.5 degrees Celsius, comfort is achieved through heat generated by occupants and electrical equipment.

Above 18.5 degrees Celsius, solar protection is necessary to avoid direct solar radiation, combined with other strategies to protect the interior of the building. As shown in Figure 2:

### 3.2 Proposal

After these analyses, a flexible modular housing proposal is presented for the Cebadas parish of the Guamote canton in the province of Chimborazo, in response to the spatial need for growth and expansion presented by the inhabitants of the sector through 6 basic guidelines that this type of housing should have: Environment, Visual, Form, Function, Bioclimatic and Materials, housing focused on four people with modular expansion capacity.

*3.2.1 Environment*

This indicator shows that the location of the project is an elemental factor in the design proposal. Therefore, it is suggested to generate a layout that takes advantage of the immediate surroundings to generate visuals and points of attraction for users, in addition to proposing collaborative and shared social spaces for a new form of community life, taking advantage of a direct connection with the main access roads and integration with paths that project a route through the design.

**Flexible modular dwelling proposal Cebadas – Ecuador:** The project, proposed in the rural-urban area of Cebadas center, carefully manages the relationship with the immediate environment, hence it is surrounded by agricultural green areas and other houses in the sector that have been urbanized over time.

**Figure 3.**
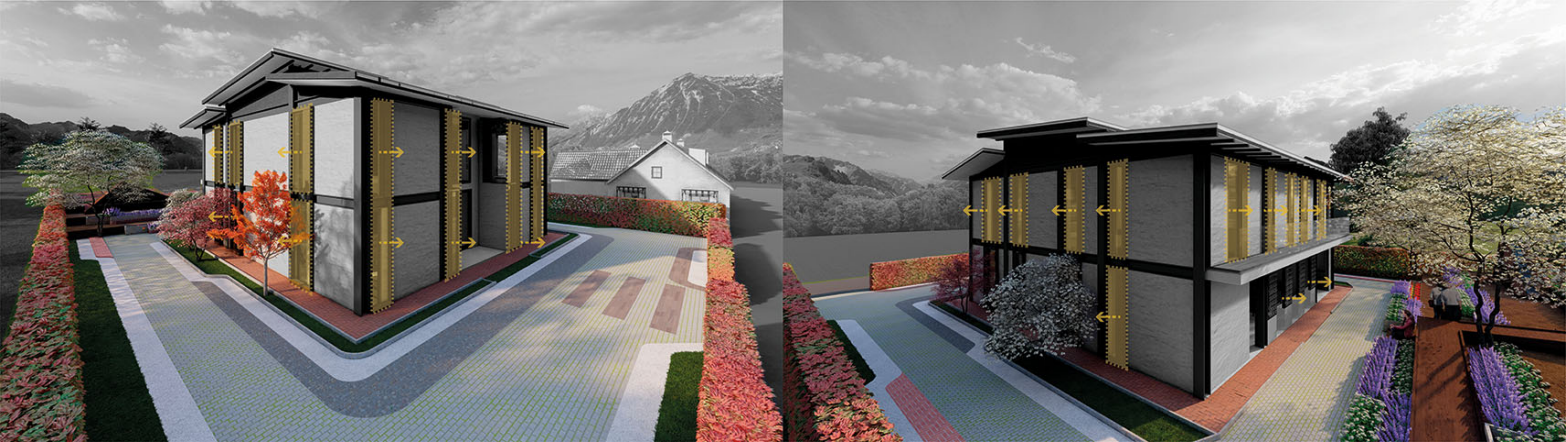
Environmental guidelines proposal applied to flexible modular dwelling Cebadas-Ecuador using ArchiCad, Lumion, Adobe Photoshop and Adobe Illustrator.

The project is located in isolation, in response to the rural environment in addition to taking advantage of the strategic location of implementation. The house responds to the natural landscape that characterizes the rural area of Cebadas, as a result the project not only seeks to integrate into the environment but also to blend in and mimic the exterior. The spaces are designed in a multifunctional way accompanied by a sensory landscape design with floor layout patterns to connect all the accesses to the rear of the project to revalue urban life with urban gardens for the sustenance of the family members. As shown in Figure 3:

*3.2.2 Visuals*

It is suggested to directly connect the surrounding environment with the architectural spaces using large windows that allow framing the visuals to appreciate the vegetation outside or at the same time, generate an interior landscape proposal, thus creating an atmosphere of tranquility for these spaces.

**Figure 4.**
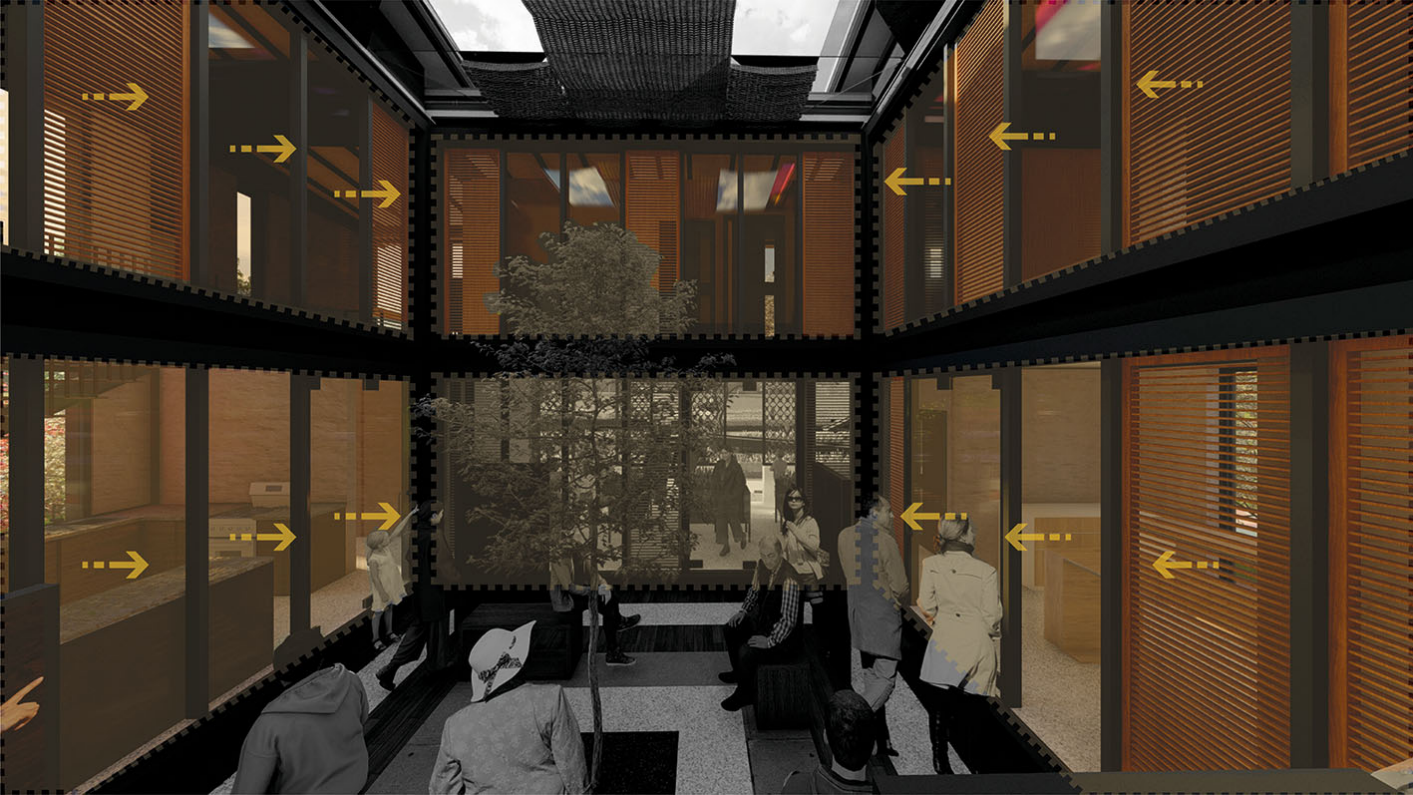
Proposal of Visual guidelines applied to flexible modular dwelling Cebadas-Ecuador using ArchiCad, Lumion, Adobe Photoshop and Adobe Illustrator.

**Flexible modular dwelling proposal Cebadas-Ecuador:** The project carefully manages the visuals of the project in an introspective way, through landscaped frames oriented to the interior garden, the barbecue area and the orchards. As shown in Figure 4:

*3.2.3 Form*

The project should start from a basic concept of modulation that generates new spaces according to the user’s needs. The formal organization of the building responds to several organizing principles with a spatial correlation.

**Figure 5.**
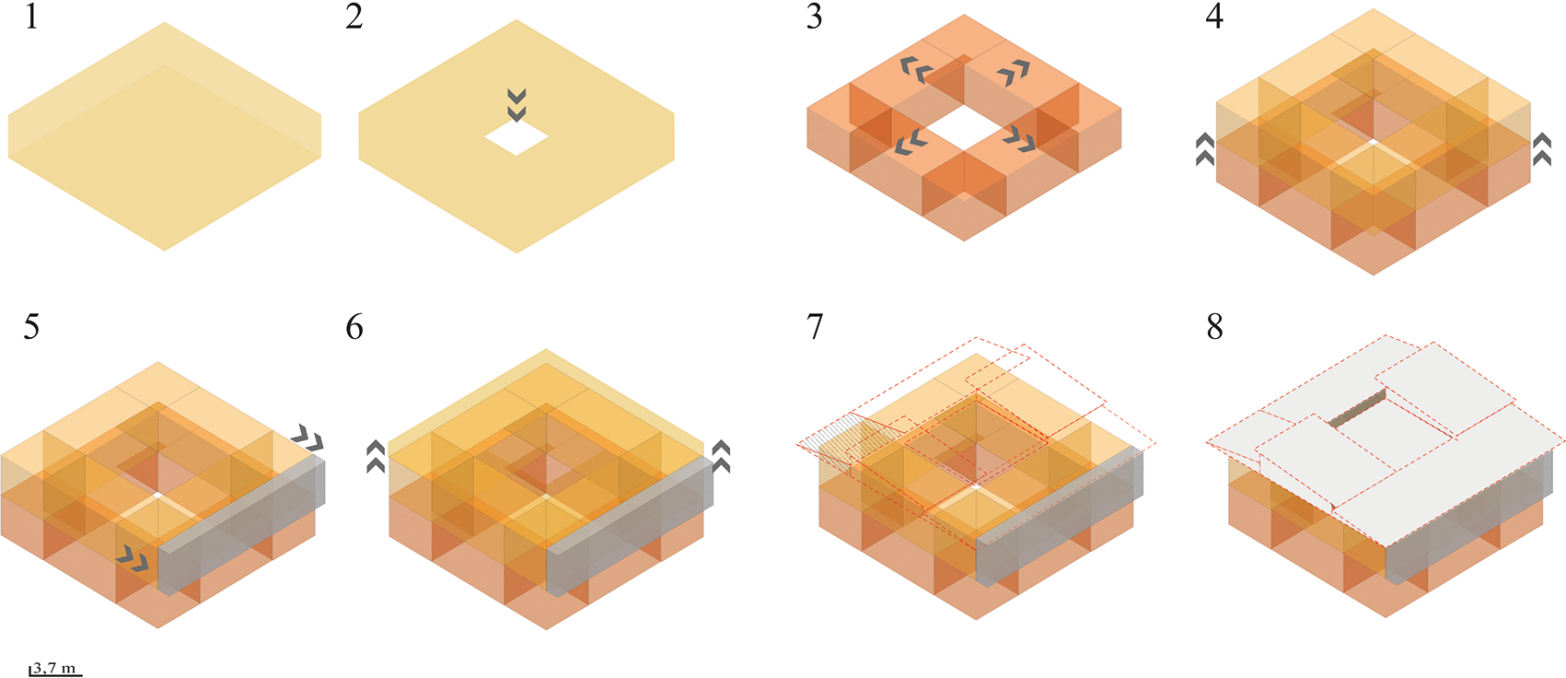
Proposal of Form guidelines applied to flexible modular dwelling Cebadas-Ecuador using Adobe Illustrator.

**Flexible modular dwelling proposal Cebadas-Ecuador:** The organization of this system begins with an abstraction in the center, thus generating an internal courtyard that becomes the articulating void of the project, this central space serves as a starting point to which the other spaces are gradually added. Following these principles of organization, half of a central space is subtracted, creating a porch-like foyer that ends in the main entrance. Expanding to the upper floor, an addition is projected at the rear that functions as a balcony, redirecting views to the surrounding landscape. Ultimately, the project seeks to break with its formal orthogonality by projecting a set of heights through sloping roofs, as shown in Figure 5:

*3.2.4 Function*

The parameter suggests posing a relationship and interconnection in the project spaces generating permeability and visual interconnection with the immediate surroundings.

**Flexible modular dwelling proposal Cebadas-Ecuador:** The project establishes an axis to connect directly with the city, allocating the first floor to communal, public and semi-public spaces, and the upper floor to private spaces, ensuring a clear separation of uses. It employs a 1 m × 1 m modulation to provide the minimum essential space, complemented by 50 cm × 50 cm micro-modules for a flexible and functional distribution. The organization starts from a central internal courtyard, one half of which is subtracted to create a porch-like foyer to the main entrance. Upstairs, a rear balcony redirects the views to the landscape.

The project breaks the orthogonality with sloping roofs, creating a set of heights.

In terms of function, as shown in Figure 5, it is organized horizontally, with a central hierarchical axis where the greenhouse is located, which distributes the areas around it. This link leads to an exhibition gallery and a workshop, creating a community social space that integrates the production and sale of handicrafts and crops, promoting local identity.

**Figure 6.**
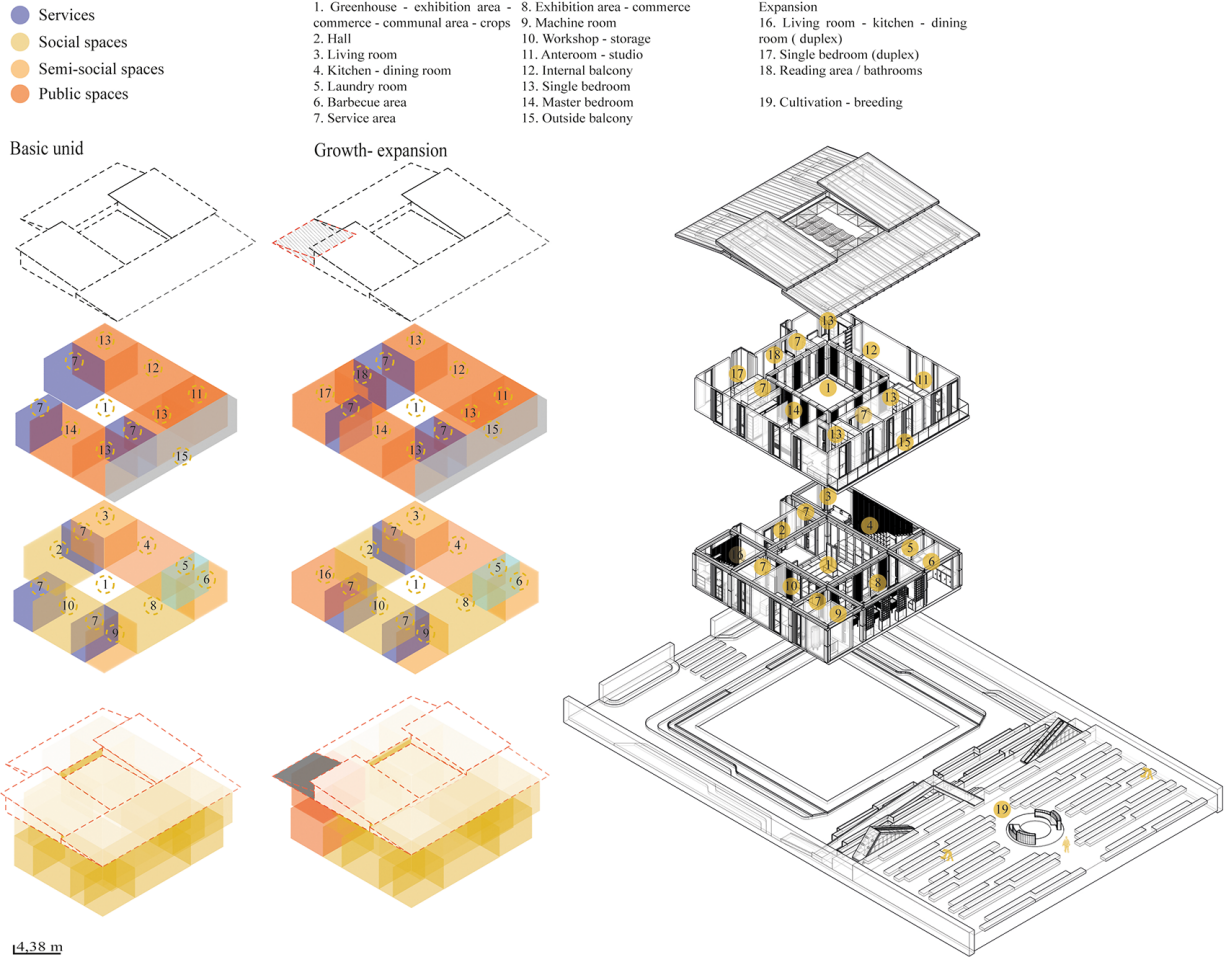
Proposed Functional Guidelines applied to the Cebadas-Ecuador flexible modular dwelling using ArchiCad, SketchUp and Adobe Illustrator.

The greenhouse acts as a key element, visually and spatially connecting the interior and exterior, functionally segmenting the house and guiding circulation in a coherent and efficient manner from the street to the rear cultivation area. The rear of the lot is transformed to enhance the landscape and encourage cultivation, recovering the identity of the sector. In addition, the design allows for the expansion of the dwelling into a duplex apartment, offering a new source of income. As shown in Figure 6:

*3.2.5 Materials*

The project should integrate materials with insulating properties, both thermal and acoustic, as these materials help the project to air condition the spaces. It also favors the use of local, sustainable materials from renewable sources.

**Figure 7.**
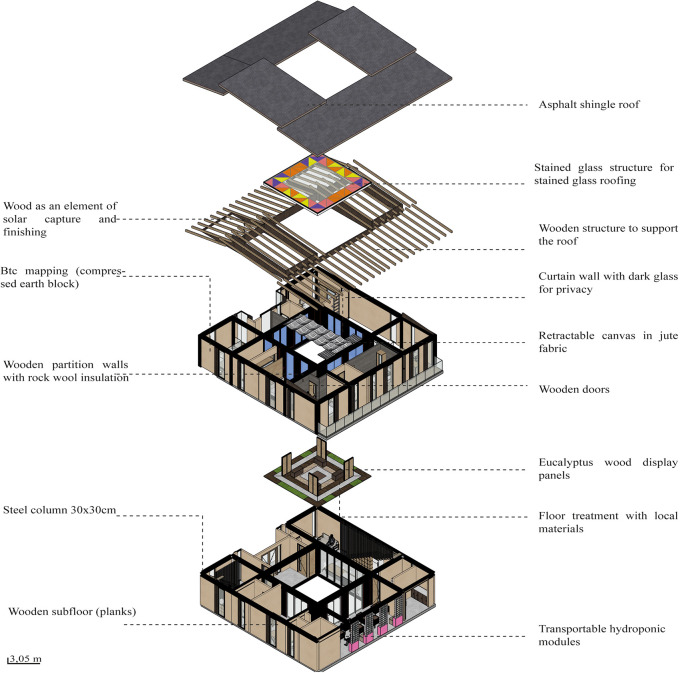
Material guidelines proposal applied to flexible modular dwelling Cebadas-Ecuador using ArchiCad, SketchUp and Adobe Illustrator.

**Flexible modular dwelling proposal Cebadas-Ecuador:** The project integrates materials with insulating properties, both thermal and acoustic, to air condition the spaces. The predominant materials are Compressed Earth Block (CEB) and steel. The CEB is used as an element of solar gain and thermal insulation to project them on most of the walls of the house. Wood, in the form of planks or boards, is mainly applied on the floor of the project to improve thermal gain as a support for the roof. The use of these local materials seeks to recover the identity and thermal characteristics of the sector’s dwellings, as shown in Figure 4. In addition, local materials are integrated with new systems such as steel, aluminum and glass. Steel and aluminum guarantee the flexibility of the spaces, while glass is used in the internal stained glass to create a suitable environment for both the landscape and the development of the internal vegetation. As shown in Figure 7.

*3.2.6 Bioclimatic*

This indicator suggests maximizing solar and heat gain through passive and active strategies.

• The project should be oriented towards the sun to obtain greater solar collection, which would allow greater thermal gain. It is important that the spatial distribution organizes the bedrooms and the social area towards the facades directly facing the sun, so that the other spaces receive indirect solar incidence.
• Gain of natural lighting in all spaces of the project.
• Thermal gain through central greenhouses that can act as multifunctional-bioclimatic-landscapes, such as internal courtyards, surrounded by social and private areas.
• Regarding ventilation, cross ventilation strategies are suggested through large windows, but avoiding the prevailing winds of the sector.
• Adaptation and use of the existing topography.
• Wind protection through the modeling of the existing topography, vegetation curtains, use of surrounding vegetation.
• Involvement of materials with high thermal inertia such as wood for the roof, handmade bricks for walls, stone for floors, etc., so that heat is transmitted to the interior of the house.
• Use of colors that allow the sun’s rays to be reflected.
• Bioclimatic strategies in the project design to increase the temperature of the building, such as the use of Trombe walls or heat traps.
• The thickness of the perimeter walls allows keeping the heat inside the building, while the internal walls of lesser thickness allow a greater use and passage of heat, maintaining an adequate temperature inside the building.
• It is suggested that the building’s electrical system be supported by a system of solar panels that capture sunlight and transform it into energy.
• Making use of the collection and recycling system as an alternative source of energy income by being able to trade recycled materials.
• Rainwater harvesting and reuse strategies, to be used as irrigation for greenhouse crops and green areas or as water reserve for the house.

**Figure 8.**
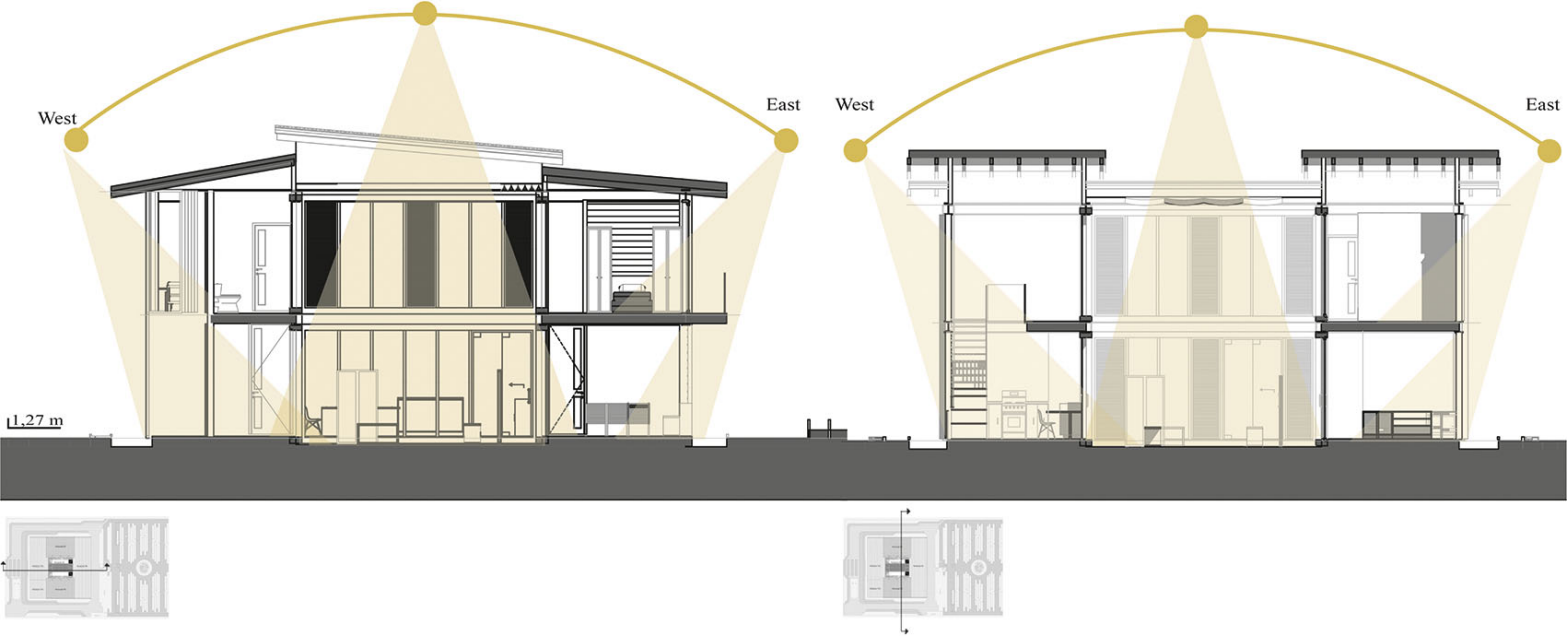
Light penetration analysis of flexible modular dwelling Cebadas-Ecuador using ArchiCad, and Adobe Photoshop.

**Figure 9.**
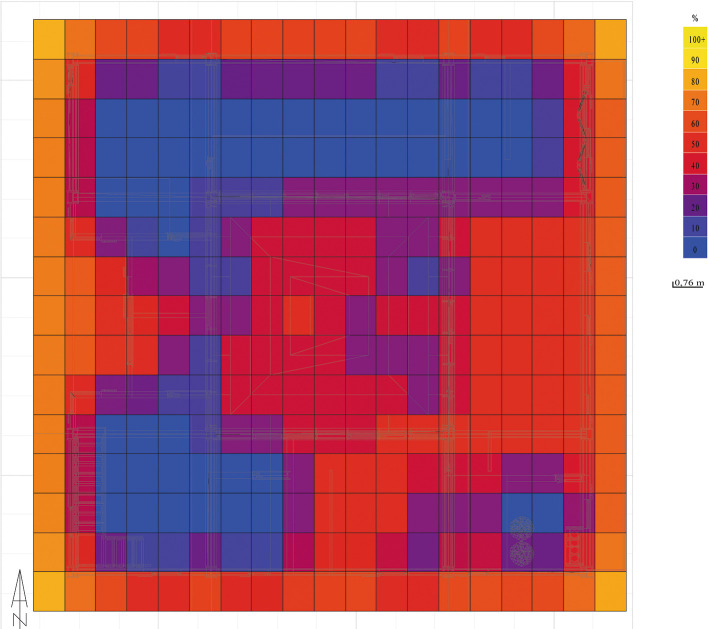
Daylighting analysis of the Cebadas-Ecuador flexible modular dwelling using Dynamic Daylighting.

**Figure 10.**
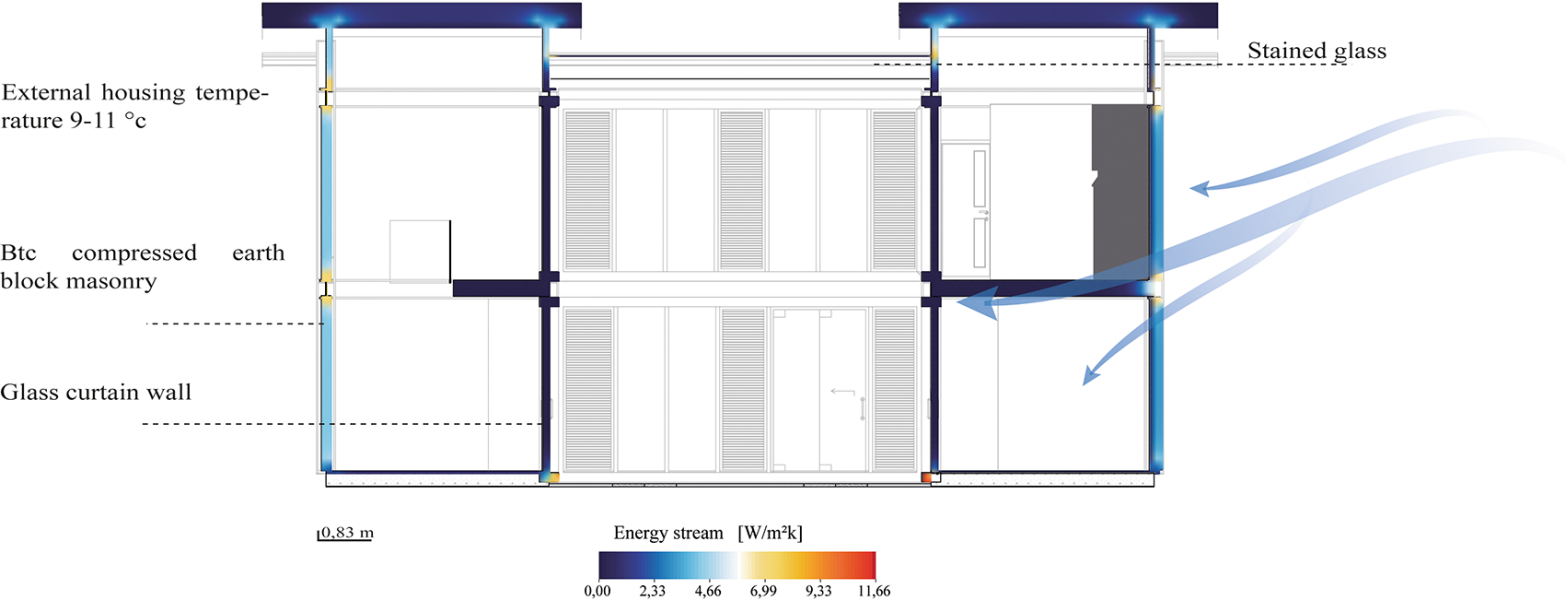
Energy Flow analysis of the Cebadas- Ecuador flexible modular dwelling using Archicad and Adobe Illustrator.

**Flexible modular dwelling proposal Cebadas-Ecuador:** The design of the lighting system focuses on providing direct light to the social and semi-social spaces of the project. Therefore, a greenhouse is projected in the central part, surrounded by curtain walls, thus allowing direct sunlight in these areas, as shown in Figure 8. This approach maximizes the entry of natural light, and also integrates the greenhouse element as a central component in the lighting distribution. Additionally, direct lighting is sought in the private spaces, specifically in the bedrooms, for which large floor-to-ceiling windows have been assigned on the side facades of the building, these windows provide an abundant entry of natural light, improving the quality of the interior lighting, as well as establishing a direct visual connection with the exterior environment. This lighting system design not only addresses practical considerations of efficient lighting, but also contributes to the thermal component of the building. As shown in Figure 8:

The analysis of natural lighting of the house is presented in percentage ranges from 0 to 100% with 0 being least illuminated spaces, where natural light does not shine directly, and 100 being the spaces that receive direct lighting. As can be seen in Figure 9 generated by Dynamic Daylighting Analysis, it is evident that the highest incidence of light is concentrated in the greenhouse, although it is at 50% because the stained glass and retractable roof strategies sift direct light to control the passage of illumination, this luminosity is distributed to the other spaces which added to the reduction in the thickness of the internal walls, makes effective the passage of heat to all spaces of the house, generating warm and well-lit spaces. The same happens on the upper floor, where the presence of openings and balconies on the level allow the entry of diffuse light, achieving uniform illumination in most of the spaces. As shown in Figure 9:

Figure 10 clearly shows that the materials used in the construction of the house have had an impact on the reduction of the energy flow, which has contributed to maintaining a constant temperature in the interior spaces.

**Figure 11.**
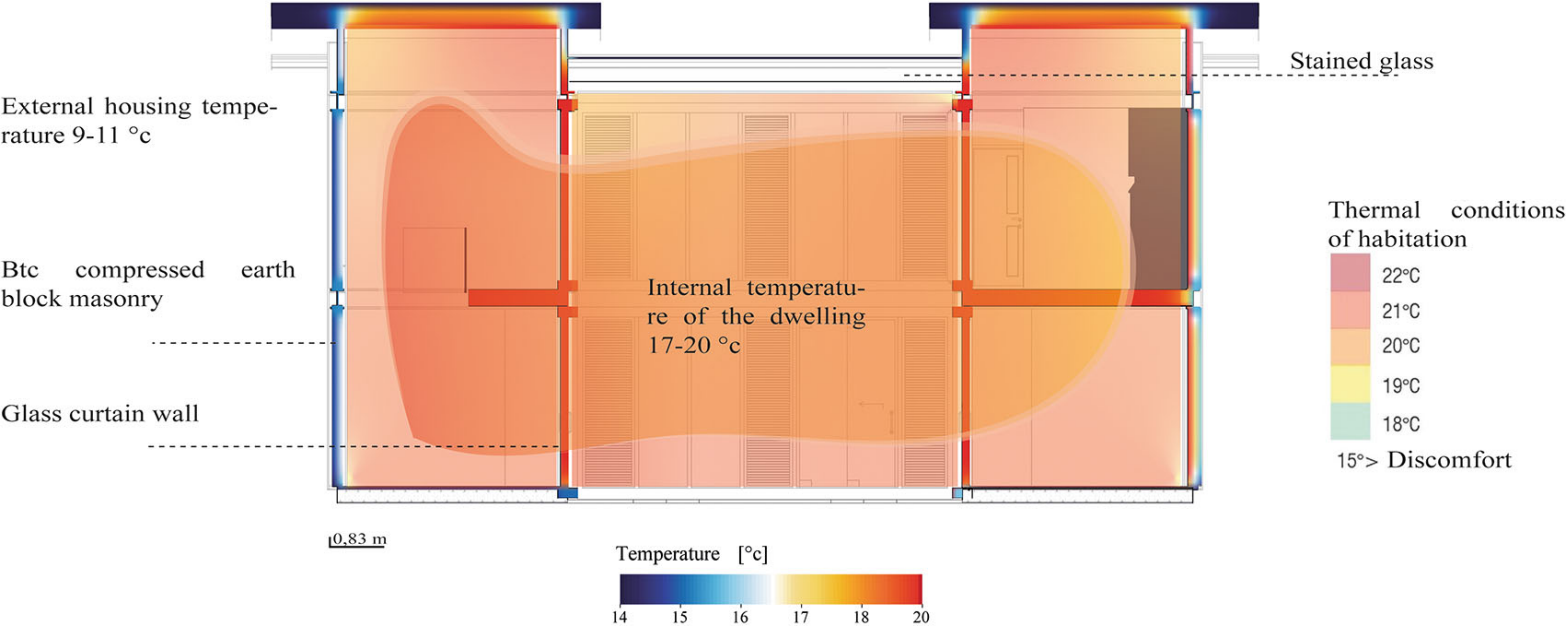
Analysis of internal temperature in the Cebadas-Ecuador flexible modular dwelling using Archicad and Adobe Illustrator.

The materials used have proven to be effective in thermal regulation by limiting heat loss or gain, hence the thermal comfort inside the house is optimal. As shown in Figure 11 the internal temperature varies between 17 and 20 degrees, numbers that are within the habitable thermal standards proposed by Olgyay, V. (1963) and Givoni, B. (1969). As shown in Figure 11:

## 4. Conclusions

• The research project on flexible modular dwelling for rural environments has explored the complex housing needs in the sector, drawing conclusions that highlight the relevance and potential of the proposal.
• Flexibility and Adaptability: The introduction of a basic housing module with the capacity for future expansion effectively addresses the changing needs of users, allowing for continuous adaptation over time and anticipating the growth of families.
• Additional Income Generation: The inclusion of a duplex apartment as part of the expansion of the project responds to the housing needs of the users, and presents an opportunity for the residents to generate additional income, thus contributing to the economy of the families.
• Community and Cultural life: The incorporation of public spaces, stores and craft exhibitions promotes community life and rescues the cultural identity of the sector, contributing to the preservation of local traditions.
• Tourism and Rural Development: The combination of landscape design with crops serves agricultural needs, promotes tourism and redefines the perception of rural life.
• Bioclimatic and Sustainability: The implementation of bioclimatic strategies, such as the central greenhouse, efficiently addresses thermal problems, improving the habitability of the house. In addition, the use of local materials and sustainability strategies such as the reuse of rainwater for crop irrigation promotes responsible management of water resources, contributing to the sustainability and efficiency of the project.

## Data Availability

Zenodo: Flexible modular dwelling for rural environments; specific case: Cebadas – Ecuador, https://doi.org/10.5281/zenodo.12785571 ( Cajamarca *et al*., 2024). Data are available under the terms of the Creative Commons Attribution 4.0 International license (CC-BY 4.0).

## Software availability

- Adobe Illustrator ( https://www.adobe.com/products/illustrator.html)/open access alternative: Inkscape ( https://inkscape.org/).
- Adobe Photoshop ( https://www.adobe.com/ec/products/photoshop/free-trial-download.html)/open access alternative: ( https://www.gimp.org/).
- ArchiCAD ( https://graphisoft.com/ec/solutions/archicad)/open access alternative: ( https://www.freecad.org/).
- Dynamic Daylighting ( https://andrewmarsh.com/software/daylight-box-web/)
- Lumion ( https://lumion.com/)/open access alternative: ( https://www.twinmotion.com/en-US ).
- Microsoft Excel ( https://www.microsoft.com/en-us/microsoft-365/excel)/open access alternative: ( https://www.libreoffice.org/discover/calc/).
- SketchUp ( https://www.sketchup.com/es)/open access alternative: ( https://www.blender.org/).
