# Supplementary material for: Flexible modular dwelling for rural environments; specific case: Cebadas – Ecuador
Source: F1000Res. 2025 Jan 17;13:845. Originally published 2024 Jul 30. [Version 2] doi: 10.12688/f1000research.154417.2 (PMC11751609; doi:10.12688/f1000research.154417.2)
Supplement: Peer review history

# Reviewer report: Reviewer response for version 2

**DOI:** 10.5256/f1000research.176417.r360149

**Published:** 2025-01-21

**Authors:** Maria del Cisne Aguirre Ullauri

**Affiliations:** 1 Universidad Católica de Cuenca, Azuay, Ecuador

We are grateful for the adjustments made; the quality of the work has improved and it will surely be useful for teachers, students and researchers in the area.

Is the case presented with sufficient detail to be useful for teaching or other practitioners?

Partly

Is the work clearly and accurately presented and does it cite the current literature?

Partly

If applicable, is the statistical analysis and its interpretation appropriate?

No

Are all the source data underlying the results available to ensure full reproducibility?

No

Are the conclusions drawn adequately supported by the results?

No

Is the background of the case’s history and progression described in sufficient detail?

Partly

Reviewer Expertise:

Architecture; historical materials; stratigraphic muraria reading; risk and management; heritage architecture

I confirm that I have read this submission and believe that I have an appropriate level of expertise to confirm that it is of an acceptable scientific standard.

## References

[1] : Vivienda modular flexible para entornos rurales; caso específico: Cebadas – Ecuador. *F1000Research* .2025;13: 10.12688/f1000research.154417.2 10.12688/f1000research.154417.2

# Reviewer report: Reviewer response for version 1

**DOI:** 10.5256/f1000research.169441.r347420

**Published:** 2024-12-28

**Authors:** Tanya Veridiana Cueva Kean Chong

**Affiliations:** 1 Universidad Estatal de Milagro, Milagro, Guayas, Ecuador

The article addresses an important issue in sustainable architecture. However, to enhance its academic and practical impact, it would be beneficial to further explore the methods, data, and graphical representations, and to provide more detailed analysis of the socioeconomic and cultural aspects of the studied context.

 The work is presented clearly and structured, using relevant citations, such as those of Garzón (2010), Sánchez (2011), Olgyay (1963), and Givoni (1969), to support its arguments on sustainability, bioclimatic design, and modularity. The literature review addresses principles of flexibility, sustainability, and adaptable design, essential for the topic discussed.

 The study design is appropriate. It combines a qualitative-quantitative approach to address both the needs of the context and the technical diagnosis. Furthermore, the research has academic merit by integrating bioclimatic, sustainable, and social criteria into the modular housing proposal for a vulnerable rural area like Cebadas.

 The methods and analyses are described in sufficient detail, including the site diagnosis, the use of tools such as bioclimatic charts and psychrometric diagrams, as well as software-assisted architectural design techniques. These elements enable other researchers to replicate or adapt the approach.

 A numerical statistical analysis is not directly mentioned; instead, bioclimatic graphs and tools (such as Olgyay and Givoni charts) are used to interpret climatic conditions. These tools are relevant to the architectural design context.

 The article mentions the use of sources such as climatic data and site characteristics, but it does not include a repository or appendix with the source data used. To ensure full reproducibility, it would be ideal to provide direct access to the supporting data

 The conclusions are well-founded on the analyses conducted and the results obtained. Flexibility, adaptability, sustainability, and income generation are ideas consistent with the data and proposals developed in the project.

**Suggestions:**

- It would be advisable to detail the numerical data used to assess the initial conditions or measure the effectiveness of the proposal.
- A deeper exploration of socioeconomic aspects is needed, as the article mentions the poverty conditions of rural housing in Cebadas, Ecuador. It would be interesting for the proposal to address issues related to the financing of these homes.
- The possible limitations of the study or challenges in implementing the proposal are not discussed, such as cultural acceptance, technical or logistical feasibility in rural contexts.
- To better communicate the key aspects of architectural design, 3D diagrams or renders would be useful.

Is the case presented with sufficient detail to be useful for teaching or other practitioners?

Yes

Is the work clearly and accurately presented and does it cite the current literature?

Yes

If applicable, is the statistical analysis and its interpretation appropriate?

Not applicable

Are all the source data underlying the results available to ensure full reproducibility?

Partly

Are the conclusions drawn adequately supported by the results?

Yes

Is the background of the case’s history and progression described in sufficient detail?

Yes

Reviewer Expertise:

Social and community architecture

I confirm that I have read this submission and believe that I have an appropriate level of expertise to confirm that it is of an acceptable scientific standard.

# Response

**Published:** 2025-01-06

**Authors:** Karina Elizabeth Cajamarca Dacto

**Affiliations:** Faculty of Engineering, Universidad Nacional de Chimborazo, Riobamba, Chimborazo Province, Ecuador

We are grateful for the valuable comments made by the reviewers, which have been carefully reviewed and incorporated into the manuscript to improve its quality and clarity. The following are the changes made:

 - List of authors: the tilde in the surname of Dany Marcelo Tasán Cruz was corrected.

 - Key words: “adaptable” was replaced by “modular” to adapt it to the context of the research.

 - Methodology: In section 2, the sections on Design and Level of research were reorganized to improve the contextualization and understanding of the object of study. A more detailed explanation of the six basic guidelines (environment, visuals, form, function, bioclimatic and materials) was incorporated.

 - Diagnosis: In section 3.1, the location of Figure No. 2 was adjusted to align it with the preceding text.

 - Proposal: In section 3.2, information on the number of inhabitants of the architectural proposal was added, as suggested by the evaluators.

 - Function: In section 3.2.1, the figure number was corrected (from 3 to 6) and its location was adjusted for consistency.

 - Figures: Graphical scales were added to figures No. 5, 6, 7, 9, 10 and 11, and solar orientation was included in figure No. 8 to improve the bioclimatic analysis.

 - Bioclimatic: In section 3.2.6, figures were reorganized for consistency between explanatory text and images.

 - Conclusions: The term “Discussion” was removed from the title of section 4 to avoid confusion.

 We are deeply grateful for the valuable suggestions of the reviewers, which were essential to optimize this article. We remain attentive to the publication process.

# Reviewer report: Reviewer response for version 1

**DOI:** 10.5256/f1000research.169441.r333945

**Published:** 2024-11-04

**Authors:** Maria del Cisne Aguirre Ullauri

**Affiliations:** 1 Universidad Católica de Cuenca, Azuay, Ecuador

The initiative of the type of work presented is welcomed. At the same time, the following observations and comments are made:

 Of form:

 1. Revise the spelling of proper nouns in their own language.

 2. Revise the naming of paragraph 2.5 considering the naming of paragraph 2.

 3.      In general, section 2 needs better structure and contextualization. For example, the macro and micro location of the object of study to enable understanding.

 4.      Revise the format of the textual citation, it is incomplete in the development of the text.

 5.      Check the punctuation in the development of the work.

 6.      Figure 2, figure 5, figure 6, figure 8 and figure 9 should be relocated according to the development of the text and the first reference given for consultation.

 7.      The use of subtitles should be reviewed to avoid those of the fourth order being more evident than those of the third order.

 8.      The typeface should be standardized. Figures have a different typeface to the text in general. The size is larger.

 9.      The wording should be revised; grammatical tenses are mixed up.

 10.   Figures should include graphic scale and orientation.

 11.   Discussion should be separated from Conclusions, necessarily.

 12.   The bibliography should be significantly improved in form and content. The sources consulted and used are limited and do not cover geographically the development of the subject and the contribution presented with the work in the world scenario. 

 13.   Technical revision of the language by a specialist is recommended.

 Background:

 1.      The summary does not present how the research work is developed, i.e. the method used and its main technical resources. The content is generic and does not match what was developed in the work being presented. The results should be expanded at a technical level in the 6 stated guidelines. Review the correspondence of the conclusions and the initial focus of the summary; the topic of tourism generates noise due to the emphasis given.

 2. It is suggested to change the keyword adaptable, it does not contribute by itself and there is another keyword (modular) that is already presented.

 3.      In the third and fourth paragraph of the introduction there is a lack of reference to authors, necessarily. Other such cases are evident throughout the document.

 4.      The introduction is weak, it does not evidence about the existing contribution to the knowledge generated by the research.

 5.      In general, section 2 needs better structure and contextualization. For example, the macro and micro location of the object of study in order to understand it.

 6.      Section 2.1 is not clear about particulars such as: type of sources consulted, period of time during which the research was carried out, method of analysis of the documents, whether qualitative or quantitative. The same applies to sections 2.2 to 2.7, which are general and do not contribute to the research process developed. 

 7.      Section 3.1 is introductory content, not a result, as stated in the work. It allows us to understand the situation of the place and the problems to be addressed, i.e. it is the state of the art, at least in the initial paragraphs.

 8.      Figure 1 refers to a specific methodology that is not indicated and explains how it was applied in the methodology section. In the following paragraph the case is repeated and extended to figure 2.

 9.      Paragraph 5 of section 3.1 needs to be reviewed for coherence and consistency of content; possibly there are contradictions.

 10.   The development of section 3.2 requires technical precision in each of the 6 components. The inclusion of renderings alone is deficient. If the context is rural, the architectural proposal may be aggressive. It is also suggested to mix conceptual descriptions with the proposal.

 11.   The proposal is not clear on certain particulars, e.g. how many people is the building designed for?

 12.   Discussion should be separated from Conclusions, necessarily. The current content is disjointed and needs more consistency.

 13.   The bibliography should be significantly improved in form and substance. The sources consulted and used are limited and do not cover geographically the development of the topic and the contribution presented with the work on the world stage.

Is the case presented with sufficient detail to be useful for teaching or other practitioners?

Partly

Is the work clearly and accurately presented and does it cite the current literature?

Partly

If applicable, is the statistical analysis and its interpretation appropriate?

No

Are all the source data underlying the results available to ensure full reproducibility?

No

Are the conclusions drawn adequately supported by the results?

No

Is the background of the case’s history and progression described in sufficient detail?

Partly

Reviewer Expertise:

Architecture; historical materials; stratigraphic muraria reading; risk and management

I confirm that I have read this submission and believe that I have an appropriate level of expertise to confirm that it is of an acceptable scientific standard, however I have significant reservations, as outlined above.

# Response

**Published:** 2024-12-28

**Authors:** Karina Elizabeth Cajamarca Dacto

**Affiliations:** Faculty of Engineering, Universidad Nacional de Chimborazo, Riobamba, Chimborazo Province, Ecuador

We have carefully reviewed the suggestions provided by the peer reviewer and would like to express our appreciation for their valuable comments. In response, we have made the necessary changes to the article to improve its clarity, content and academic rigor. Likewise, changes have been implemented in the content of the article to ensure that all modifications are consistent with the observations made, improving the quality of the work presented.

# Response

**Published:** 2025-01-04

**Authors:** Karina Elizabeth Cajamarca Dacto

**Affiliations:** Faculty of Engineering, Universidad Nacional de Chimborazo, Riobamba, Chimborazo Province, Ecuador

Agradecemos los valiosos comentarios realizados por los revisores. Todas las sugerencias y comentarios han sido cuidadosamente revisados e incorporados al manuscrito, con el objetivo de mejorar su calidad y claridad. Los siguientes son los cambios realizados:

 - Sección de listado de autores: faltas de ortografía, tilde en el apellido de uno de los autores, Dany Marcelo Tasán Cruz, fueron corregidos.

 - Sección de palabras clave: La palabra clave adaptable se ha cambiado a modular porque se adapta mejor al contexto de la investigación.

 - Apartado Método: En el apartado 2 de Métodos, se han modificado los apartados correspondientes al Diseño de la investigación y al nivel de investigación con el fin de conseguir una mejor estructura, contextualización y comprensión del objeto de estudio. Estos cambios permiten una explicación más clara y precisa de la macro y micro localización del objeto de estudio. Asimismo, se ha cambiado el nombre de la sección 'Método' por el de 'Metodología', ya que el subtítulo tenía el mismo nombre. En cuanto a su contenido, se ha incorporado una explicación más clara de las seis pautas básicas que deben cumplir este tipo de viviendas: medio ambiente, visuales, forma, función, bioclimática y materiales. Con ello se pretende aportar mayor claridad a la metodología de esta investigación y al proceso que conduce a la propuesta arquitectónica.

 - Apartado Diagnóstico: En el apartado 3.1, correspondiente al diagnóstico del parámetro de resultados, se ha ajustado la ubicación de la figura N 2 para alinearla con el texto anterior, consiguiendo así un orden lógico y coherente del texto explicativo seguido de la figura.

 - Sección de Propuesta: En el apartado 3.2, correspondiente al parámetro de resultados, se ha incorporado la información sobre el número de personas que habitarán la propuesta arquitectónica, atendiendo a la sugerencia de uno de los pares evaluadores.

 - Función de sección: En el apartado 3.2.1, correspondiente al parámetro de la propuesta, se ha corregido el número de figura, cambiándolo de 3 a 6, para garantizar la coherencia con la materia. Asimismo, se ha ajustado la ubicación de la figura número 6 para alinearla con el texto anterior, consiguiendo así un orden lógico y coherente del texto explicativo seguido de la figura.

 - Figuras: Se ha modificado el contenido de las figuras N 5, 6, 7, 9, 10 y 11, añadiendo una escala gráfica que permite la representación visual y proporcional de la relación entre las dimensiones de un objeto o espacio en el mundo real y su representación en un plano o dibujo. Esto permite al observador del dibujo o plano interpretar correctamente las medidas y proporciones, facilitando su comprensión y análisis. Ya que la escala gráfica es especialmente útil en planos arquitectónicos, mapas y diagramas, ya que proporciona una referencia clara de tamaño y distancia. Y en cuanto a la figura N 8, se ha modificado su contenido, añadiendo la orientación solar para entender mejor el análisis bioclimático objeto de la presentación.

 - Bioclimática: En el apartado 3.2.6, correspondiente al parámetro de la propuesta, se ha ajustado la ubicación de las figuras del subapartado (figuras nº 8, 9, 10 y 11) para alinearlas con el texto anterior, consiguiendo así un orden lógico y coherente en el que al texto explicativo le siguen las figuras.

 - Conclusiones/Discusión: En la sección 4 del documento, en el título combinado, se ha eliminado el término "Discusión" porque genera confusión en relación con el tema de investigación.

 Estos son los cambios incorporados en la nueva versión del documento. Estamos profundamente agradecidos por todas las valiosas sugerencias proporcionadas por los revisores, que han sido fundamentales para mejorar el contenido de este artículo. Permanecemos atentos al proceso de actualización y al avance del respectivo proceso de publicación. Saludos y gracias.

# Response

**Published:** 2025-01-04

**Authors:** Karina Elizabeth Cajamarca Dacto

**Affiliations:** Faculty of Engineering, Universidad Nacional de Chimborazo, Riobamba, Chimborazo Province, Ecuador

We are grateful for the valuable comments made by the reviewers. All suggestions and comments have been carefully reviewed and incorporated into the manuscript, with the aim of improving its quality and clarity. The following are the changes made:

 - List of authors section: spelling mistakes, tilde in the surname of one of the authors, Dany Marcelo Tasán Cruz, were corrected.

 - Key words section: The key word adaptable has been changed to modular because it is better suited to the context of the research.

 - Method section: In section 2 of Methods, the sections corresponding to the Design of the research and the level of research have been modified in order to achieve a better structure, contextualization and understanding of the object of study. These changes allow a clearer and more precise explanation of the macro and micro location of the object of study. Likewise, the name of the section 'Method' has been changed to 'Methodology', since the subtitle had the same name. As for its content, a clearer explanation of the six basic guidelines that this type of housing must comply with has been incorporated: environment, visuals, form, function, bioclimatic and materials. This is intended to provide greater clarity to the methodology of this research and the process leading to the architectural proposal.

 - Diagnosis Section: In section 3.1, corresponding to the diagnosis of the results parameter, the location of figure N 2 has been adjusted to align it with the preceding text, thus achieving a logical and coherent order of explanatory text followed by the figure.

 - Proposal Section: In section 3.2, corresponding to the results parameter, the information on the number of people who will inhabit the architectural proposal has been incorporated, attending to the suggestion of one of the peer reviewers.

 - Section Function: In section 3.2.1, corresponding to the proposal parameter, the figure number has been corrected, changing it from 3 to 6, to ensure consistency with the subject matter. Also, the location of figure number 6 has been adjusted to align it with the preceding text, thus achieving a logical and coherent order of the explanatory text followed by the figure.

 - Figures: The content of figures N 5, 6, 7, 9, 10 and 11 has been modified, adding a graphic scale that allows the visual and proportional representation of the relationship between the dimensions of an object or space in the real world and its representation in a plan or drawing. This allows the observer of the drawing or plan to correctly interpret the measurements and proportions, facilitating its understanding and analysis. Since the graphic scale is especially useful in architectural plans, maps and diagrams, as it provides a clear reference of size and distance. And as for figure N 8, its content has been modified, adding the solar orientation to better understand the bioclimatic analysis object of the presentation.

 - Bioclimatic: In section 3.2.6, corresponding to the proposal parameter, the location of the figures in the subsection (figures No. 8, 9, 10 and 11) has been adjusted to align them with the preceding text, thus achieving a logical and coherent order in which the explanatory text is followed by the figures.

 - Conclusions/Discussion: In section 4 of the document, in the combined title, the term “Discussion” has been removed because it generates confusion in relation to the research topic.

 These are the changes incorporated in the new version of the paper. We are deeply grateful for all the valuable suggestions provided by the peer reviewers, which have been fundamental to improve the content of this article. We remain attentive to the updating process and to the progress of the respective publication process. Greetings and thanks.

# Response

**Published:** 2025-01-06

**Authors:** Karina Elizabeth Cajamarca Dacto

**Affiliations:** Faculty of Engineering, Universidad Nacional de Chimborazo, Riobamba, Chimborazo Province, Ecuador

Dear Editor, I enclose the text in the requested number of words: 

 We are grateful for the valuable comments made by the reviewers, which have been carefully reviewed and incorporated into the manuscript to improve its quality and clarity. The following are the changes made:

 - List of authors: the tilde in the surname of Dany Marcelo Tasán Cruz was corrected.

 - Key words: “adaptable” was replaced by “modular” to adapt it to the context of the research.

 - Methodology: In section 2, the sections on Design and Level of research were reorganized to improve the contextualization and understanding of the object of study. A more detailed explanation of the six basic guidelines (environment, visuals, form, function, bioclimatic and materials) was incorporated.

 - Diagnosis: In section 3.1, the location of Figure No. 2 was adjusted to align it with the preceding text.

 - Proposal: In section 3.2, information on the number of inhabitants of the architectural proposal was added, as suggested by the evaluators.

 - Function: In section 3.2.1, the figure number was corrected (from 3 to 6) and its location was adjusted for consistency.

 - Figures: Graphical scales were added to figures No. 5, 6, 7, 9, 10 and 11, and solar orientation was included in figure No. 8 to improve the bioclimatic analysis.

 - Bioclimatic: In section 3.2.6, figures were reorganized for consistency between explanatory text and images.

 - Conclusions: The term “Discussion” was removed from the title of section 4 to avoid confusion.

 We are deeply grateful for the valuable suggestions of the reviewers, which were essential to optimize this article. We remain attentive to the publication process.
